# Decentralising drug-resistant TB treatment initiation services

**DOI:** 10.5588/ijtldopen.25.0412

**Published:** 2025-12-10

**Authors:** N. Murphy-Okpala, C. Eze, I.N. Orabueze, I. Ezenwosu, C. Nwafor, N. Ekeke, O. Ezeakile, J.N. Chukwu, S. Matiku, A. Meka, M. Njoku, B. Kirubi, F. Iyama, E. Ossai, O. Chijioke-Akaniro, J. Creswell

**Affiliations:** 1 Department of Programs, RedAid Nigeria, Enugu, Nigeria;; 2 Department of Medical Microbiology, Faculty of Basic Clinical Sciences, College of Medicine, University of Nigeria Ituku-Ozalla Campus, Enugu, Nigeria;; 3 Department of Community Medicine, University of Nigeria Teaching Hospital, Ituku-Ozalla, Nigeria;; 4 New Dimension Consulting, Dar es Salaam, Tanzania;; 5 Stop TB Partnership, Geneva, Switzerland;; 6 Department of Community Medicine, Faculty of Health Sciences, Ebonyi State University, Abakaliki, Nigeria;; 7 National TB, Leprosy and Buruli Ulcer Control Program, Abuja, Nigeria.

**Keywords:** tuberculosis, pre-treatment loss-to-follow-up, DR-TB, treatment enrolment

## Abstract

**BACKGROUND:**

Drug-resistant TB (DR-TB) care coordination in Nigeria remains largely centralised, negatively impacting pre-treatment loss-to-follow-up (PTLTFU) and time-to-treatment initiation. We piloted a multi-faceted intervention and documented how the decentralisation of DR-TB services affected treatment enrolment and time-to-treatment initiation.

**METHODS:**

A quasi-experimental study was conducted in Southern Nigeria. Multi-level intervention consisting of eight components was implemented in the intervention states over a 15-month period. Data were collected using desk review proformas and analysed.

**RESULTS:**

At baseline, comparable proportions of people with DR-TB initiated treatment (χ^2^ = 3.150, *P* = 0.076). Following decentralisation, a higher proportion (79.1%) of diagnosed persons with DR-TB in the intervention states were enrolled into treatment compared with the control states, 66.0% (χ^2^ = 15.232, *P* < 0.001). There was a significant reduction in PTLTFU in the intervention states from 39.5% to 20.9% (*P* < 0.001) while PTLTFU increased from 31.9% to 34.0% (*P* = 0.689) in the control states. The median time-to-treatment initiation decreased from 17 days (interquartile range [IQR]: 10.0–32.0) at baseline to 14 days (IQR: 9.0–25.2) post-intervention. In the control states, median time-to-treatment initiation decreased from 21 days (IQR: 13.0–35.3) at baseline to 15 days (8.0–36.0) post-intervention.

**CONCLUSION:**

Decentralising DR-TB services significantly reduced the diagnosis–enrolment gap and time-to-treatment initiation. Our findings provide contextual evidence for the expansion of decentralised services in Nigeria.

In 2022, the WHO estimated that 410,000 people developed multidrug-resistant or rifampicin-resistant TB (MDR/RR-TB) worldwide. Of these people with drug-resistant TB (PwDRTB), only 42.8% were enrolled on treatment, meaning about three in five people who needed treatment for drug-resistant TB (DR-TB) were missed.^1^ Nigeria is among the 10 countries that accounted for 70% of the global gap in the estimated number of people who developed MDR/RR-TB and the number of people diagnosed and enrolled for treatment in 2022.^1^ Among the 410,000 estimated MDR/RR-TB cases globally, 176,650 (43%) people were enrolled on treatment.^1^ In Nigeria, only 2,975 (14%) of the estimated 21,000 DR-TB cases were notified in 2021, and 26% of them experienced pre-treatment loss-to-follow-up (PTLTFU).^2^ This diagnosis–enrolment gap adversely affected the achievement of the first United Nations High-Level Meeting’s target of identifying and enrolling 1.5 million PwDRTB on treatment by 2022 globally.^3^

Traditionally, DR-TB treatment initiation services in Nigeria have been centralised with management at the state level, which limits availability and access to DR-TB care and inadvertently prolongs time-to-treatment initiation. Decentralisation of TB services has been highlighted as an important strategy to bridge the widening gap in access to TB care.^4^ Studies have shown decentralised care was more likely than centralised care to lead to reduced PTLTFU, reduced cost, shorter time-to-treatment initiation, and improved treatment success rate.^5–7^ Furthermore, the decentralisation of care approach in DR-TB management is considered vital and cost-effective, especially in low-resource settings.^8^ RedAid Nigeria implemented the TB REACH Wave 9 project and measured the effect of decentralising DR-TB services under routine programming conditions. TB REACH is an initiative of Stop TB Partnership, which funds innovative solution for TB case detection. Our pilot project aimed to reduce PTLTFU for PwDRTB and used a comprehensive qualitative assessment identified enablers and barriers which shaped the implementation of decentralised DR-TB services, demonstrating its ease and advantages over a centralised approach.^9^

This study aimed to evaluate the impact of decentralised DR-TB services on the proportion of PwDRTB enrolled in care as well as their time-to-treatment initiation, while providing evidence to guide policy and practice in expanding these services in Nigeria.

## METHODS

This quasi-experimental study was conducted in Akwa-Ibom and Oyo states in southern Nigeria. We used the standard TB REACH approach to measure the impact of interventions on the population-level linkage to treatment compared with historical and contemporary controls.^10^ Two epidemiologically similar locations (Delta and Edo states) were selected as controls. The total population size of the study area is 16,076,359 in 2022 as projected from 2006 census.^11^ The interventions aimed to reduce the DR-TB diagnosis–enrolment gap by piloting decentralisation of DR-TB services under routine programmatic conditions. Routine TB programmes in both Akwa-Ibom and Oyo states are funded by the National Tuberculosis, Leprosy and Buruli Ulcer Control Program (NTBLCP), Global Fund (GF), as well as the United States Agency for International Development Local Organizations Networks (USAID TB LON) project grants anchored by KNCV Nigeria in Akwa-Ibom and the Institute for Human Virology Nigeria (IHVN) in Oyo state. The Oyo State TB Control Programme further receives technical support from Damien Foundation Belgium (DFB Nigeria). In Oyo and Akwa-Ibom, there are 671 and 538 directly observed treatment, short-course (DOTS) facilities, respectively, and 28 GeneXpert machines (Oyo, 15 and Akwa-Ibom, 13). Akwa-Ibom state has one DR-TB treatment centre and uses both the south-east and south-south zonal reference laboratories in Amachara, Abia state, and Port Harcourt, Rivers state, whereas Oyo state has two DR-TB treatment centres and the south-west zonal reference laboratory. During the 3 years (2019–2021) preceding our intervention, the proportion of PwDRTB enrolled on treatment was 70%, 52%, and 56% in the intervention states and 63%, 56%, and 67% in the control states, respectively.

### Interventions for decentralised DR-TB treatment initiation services

Under the TB REACH Wave 9 project, several multi-level interventions were strategically designed to address identified gaps in the DR-TB care pathway in Nigeria from the point of laboratory confirmation of a DR-TB diagnosis up to treatment initiation. The package of different interventions designed to synergistically reduce PTLTFU which were implemented by the existing health workers and relevant personnel involved in the routine programmatic management of DR-TB are as follows (see Supplementary Data for detailed description):

1. Devolving DR-TB diagnosis notification to include local government TB supervisors (TBLS) (using WhatsApp) as well as to the person with DR-TB
2. Working with community-based organisations to improve tracing of PwDRTB through output-based financial incentives
3. Improving counselling of PwDRTB through introduction of structured pre-treatment counselling and team counselling with DR-TB survivors
4. Decentralising baseline investigations to more (pre-qualified) peripheral laboratories
5. Providing transport support to PwDRTB for baseline investigations
6. Employing mobile connectivity solutions to improve turnaround time of baseline investigations using Unstructured Supplementary Service Data
7. Decentralising treatment initiation to the local government area level
8. Engagement of a state (volunteer) liaison officer to coordinate state-wide activities

A series of trainings for capacity building was conducted for all cadres of frontline health workers who implemented the interventions. This was followed by close, supportive supervision and mentoring throughout the implementation process. The schematic diagram (Figure) illustrates the standard care pathway to DR-TB treatment initiation and the project-adapted patient care pathway with decentralised DR-TB treatment initiation services as operationalised in the project’s intervention states.

**Figure.**
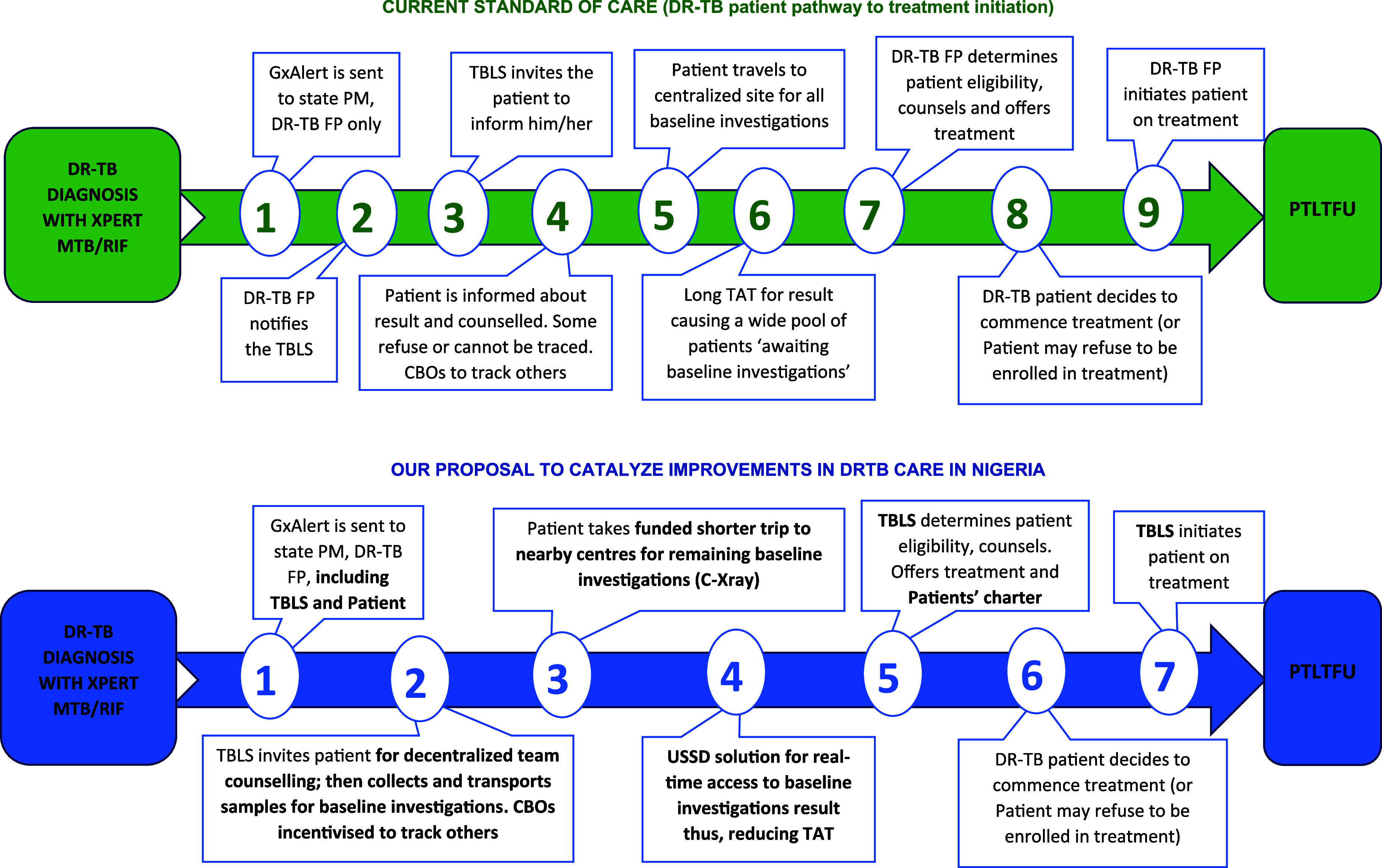
Pictorial depiction of the patient care pathway in centralised standard of care versus proposed multi-level interventions for decentralisation of DR-TB services to reduce pre-treatment loss-to-follow-up. PM = state programme manager; DR-TB FP = state DR-TB focal person; TBLS = TB Local Government Supervisor; C-Xray = chest X-ray; ECG = electrocardiogram; USSD = unstructured supplementary service data; TAT = turnaround time; PTLTFU = pre-treatment loss-to-follow-up; PPE2 = proportion of persons enrolled in 2 weeks.

### Recruitment technique

From 1 April 2022 to 30 June 2023, all individuals diagnosed with laboratory-confirmed MDR/RR-TB in the four intervention and control states were eligible for inclusion. Using routine surveillance data of all notified DR-TB cases, a whole population study was carried out.

### Data collection

Desk review proformas were used to collect retrospective information from the state routine TB/DR-TB registers to document relevant patient characteristics such as age, gender, date of diagnosis, date of treatment initiation, and enrolment status (enrolled or not enrolled).

### Patient and public involvement

Although patient opinions contributed to the overall project design, patients and the public were not directly involved in this research as it was largely a desk review of routine programme data within the project duration.

### Enrolment in treatment

The primary outcome of the study was the proportion of PwDRTB enrolled in treatment at both project intervention and control states. Operationally, we defined some as PTLTFU if they did not start on DR-TB treatment by 30 June 2023, which was the end of 15-months intervention implementation.

### Time-to-treatment initiation

We measured the number of days accrued from the date of GeneXpert result of rifampicin resistance (diagnosis) as documented on the laboratory form until the DR-TB treatment initiation. The median time-to-treatment initiation was calculated for both intervention and control states.

### Statistical analysis

The International Business Machine-Statistical Product and Service Solutions (IBM-SPSS) version 25.0 was used to analyse the data. Demographic characteristics were analysed using frequencies and proportions for categorical variables and mean/standard deviation or median with corresponding interquartile range (IQR) for continuous variables as appropriate. A 5-quarter historic trend of DR-TB enrolment was analysed from routine surveillance TB data in the intervention and control states. To determine the effectiveness of decentralising DR-TB treatment initiation services, we compared proportion of PwDRTB enrolled on treatment before and after the intervention between the project and control states with the Pearson χ^2^ test while the McNemar χ^2^ test was used to compare the outcome before and after the intervention within the groups.

### Ethical statement

Ethical approval was granted by the National Health Research Ethics Committee, Nigeria, NHREC/01/01/2007-15/06/2023. All desk review data analysed were collected as part of routine diagnosis and treatment and as such did not require individual participants’ consent. The study was also duly registered with a WHO-approved primary registry, Pan African Clinical Trial Registry PACTR202309676675265, https://pactr.samrc.ac.za/TrialDisplay.aspx?TrialID=25838.

## RESULTS

Table 1 shows baseline and post-intervention comparison of treatment initiation among PwDRTB in the intervention and control states. At baseline, comparable proportions of individuals newly diagnosed with DR-TB in the intervention states (60.5%) and the control states (68.1%) were enrolled into treatment for DR-TB (χ^2^ = 3.150, *P* = 0.076). After the intervention period, a statistically significant higher proportion (79.1%) of PwDRTB in the intervention states were enrolled into treatment when compared with those in the control states, 66.0% (χ^2^ = 15.232, *P* < 0.001). Thus, a significant reduction in the PTLTFU by half (from 39.5% to 20.9%) was observed in the intervention group.

**Table 1.** Baseline and post-intervention comparison of treatment enrolment among PwDRTB in the intervention and control states.

Variable	Intervention states, n = 387 (%)	Control states, n = 188 (%)	χ^2^	*P* value
Baseline				
DR-TB treatment enrolment				
Yes	234 (60.5)	128 (68.1)	3.150	0.076
No	153 (39.5)	60 (31.9)		
Post-intervention				
DR-TB treatment enrolment	334 (79.1)	151 (66.0)	15.232	<0.001
Yes	88 (20.9)	73 (34.0)		
No				

DR-TB = drug-resistant TB; PwDRTB = people with DR-TB.

Table 2 shows within-group comparison of treatment initiation among PwDRTB in the intervention and control groups. A significantly higher proportion of PwDRTB in the intervention states (79.1%) were initiated into DR-TB treatment post-intervention when compared with baseline levels (60.5%) (McNemar χ^2^, *P* < 0.001). Comparable proportions of PwDRTB in the control states were initiated into DR-TB treatment post-intervention (66.0%) and at baseline period (68.1%) (McNemar χ^2^, *P* = 0.689).

**Table 2.** Within-group comparison of treatment enrolment among PwDRTB in the intervention and control groups.

Variable	Baseline, n = 387 (%)	Post-intervention, n = 422 (%)	Test	*P*-value statistic
Intervention states				
DR-TB treatment enrolment				
Yes	234 (60.5)	334 (79.1)	McNemar χ^2^	*P* < 0.001
No	153 (39.5)	88 (20.9)		
Control states				
DR-TB treatment enrolment				
Yes	128 (68.1)	151 (66.0)	McNemar χ^2^	*P* = 0.689
No	60 (31.9)	73 (34.0)		

DR-TB = drug-resistant TB; PwDRTB = people with DR-TB.

The median time-to-treatment is described in Table 3, which decreased from 17 days (IQR = 10–32 days) to 14 days (IQR = 9.0–25.2) among the intervention group. For the control group, the median time-to-treatment also decreased from 21 days (IQR = 13.0–35.3) to 15 days (IQR = 8.0–36.0). Furthermore, a comparison of treatment initiation within 14 days in intervention and control states shows that a higher proportion of PwDRTB in the intervention group (52.1%) were initiated on treatment within 14 days when compared with those in the control group (48.3%), but the difference in proportions was not found to be statistically significant (χ^2^ = 0.586, *P* = 0.444).

**Table 3.** Baseline and post-intervention comparison of time-to-treatment initiation in the intervention and control states.

Variable	Intervention states	Control states	χ^2^	*P* value
Baseline				
Time-to-treatment (days)				
Median (IQR)	17 (10.0–32.0)	21 (13.0–35.3)		
Initiated on DR-TB treatment within 14 days	n = 234 (%)	n = 128 (%)		
Yes	96 (41.0)	39 (31.0)	3.546	0.060
No	138 (59.0)	87 (69.0)		
Post-intervention				
Time-to-treatment (days)				
Median (IQR)	14 (9.0–25.2)	15 (8.0–36.0)		
Initiated on DR-TB treatment within 14 days	n = 334 (%)	n = 151 (%)		
Yes	174 (52.1)	73 (48.3)	0.586	0.444
No	160 (47.9)	78 (51.7)		

DR-TB = drug-resistant TB; IQR = interquartile range.

## DISCUSSION

This study provides insight into the trend of DR-TB enrolment under the routine centralised system, and the impact of decentralised DR-TB services on enrolment of PwDRTB, as well as the time-to-treatment initiation. While globally the official numbers of PwDRTB detected and enrolled are almost the same, this is likely due to under-reporting of laboratory diagnosis and waiting for people to be started on treatment. Several studies have documented PTLTFU for people with both drug-sensitive TB (DS-TB) and DR-TB, and there are likely more challenges faced by people starting DR-TB treatment.^12,13^ At baseline, we documented 40% PTLTFU in the intervention areas in our study. At baseline, both the intervention and control arms had similar enrolment rates for PwDRTB. However, after the implementation of decentralised DR-TB services, the proportion of PTLTFU was reduced by 50% and a significantly higher proportion of individuals (79.1%) were initiated into treatment in the intervention group when compared with the routine centralised treatment in the control group (66%). The increase in the proportion of people enrolled in DR-TB care following the decentralisation of TB services is comparable with findings by Evans et al.,^14^ who reported a 17.2% increase in the proportion of people initiating DR-TB treatment following the decentralisation of DR-TB services in South Africa.

A recent study from India used a cut-off of 14 days for defining PTLTFU among people with DS-TB.^15^ In our setting, for PwDRTB even after the intervention, almost half (48.3%) of PwDRTB did not start treatment before 14 days. Studies that document the barriers to DR-TB treatment initiation are needed. The proportion of persons enrolled into treatment within 14 days of diagnosis also rose from 41% at baseline to 52% post-intervention. Similarly, Scheffer et al.^16^ reported that in Florianopolis, decentralised TB treatment resulted in a higher proportion of patients initiating therapy early. However, Iruedo and Pather found that in contrast to decentralised care, centralised services had a greater proportion of persons initiating TB treatment early.^17^ The high rate of DR-TB enrolment following interventions for decentralisation in our study may be because of the reduced cost to access services and greater retention of patients that occurs when care is given locally.^5,18^

The median time-to-treatment initiation in the intervention group decreased from 17 days at baseline to 14 days post-intervention. This observation is in line with findings in a study conducted in Johannesburg which reported reduced time-to-treatment initiation following decentralisation, as median time-to-treatment initiation decreased from 33 to 14 days.^14^ Similarly, in KwaZulu-Natal, Loveday et al.^19^ reported that median time-to-treatment initiation was significantly shorter at decentralised sites compared with the centralised hospital at 72 versus 93 days, respectively. Conversely, Iruedo and Pather found that in OR Tambo district in South Africa, although median time-to-treatment initiation varied according to the initiating facility, time-to-treatment was essentially the same for centralised and decentralised clinics.^17^ Chen et al.^20^ and Evans et al.^14^ found that expansion of Xpert MTB/RIF and decentralisation of care setting resulted in marked reduction in time-to-treatment initiation. In our study, the decentralisation of laboratory services for conducting baseline investigations before the commencement of treatment may have contributed to the improved time-to-treatment initiation we observed. In the control states, we observed that time-to-treatment initiation decreased from 21 days at baseline to 15 days in the study’s post-intervention period. This notable improvement in median time-to-treatment initiation may be due to other programmatic or external factors that may have been addressed within the study period. For instance, the new all-oral shorter DR-TB regimens were recently introduced into Nigeria’s TB programme, which likely contributed to increasing patient acceptance. Our findings replicate experiences of other low- and middle-income countries like Bangladesh,^21^ Pakistan,^22^ and Zambia,^23^ where decentralisation was associated with improved treatment enrolment and reduced treatment delays. Comparisons reveal that decentralisation success depends heavily on robust planning, adequate resources, and effective implementation as poorly implemented decentralisation led to reduced quality of care in some settings within Pakistan.^22^

Limitations of this study include that retrospective information was collected from the state’s routine DR-TB registers. As such, we were unable to confirm whether all laboratory-confirmed PwDRTB were documented in the state registers and, thus, included in the analysis. With this, there’s a chance that this may have contributed to an underestimation of laboratory-confirmed DR-TB cases in the states, but this was true for both intervention and control areas. Our pragmatic study design was unable to identify which component of the multiple interventions we used had more or less impact on PTLTFU. A study from South Africa looked at the impact of Xpert testing on PTLTFU and concluded that improvements were not due to laboratory tests but other programmatic improvements which were also not defined.^13^ Finally, intervention and control states were selected purposefully and might not be representative of other Nigerian states. However, the various State TB Programs share similar challenges and logistic problems concerning DR-TB service delivery; hence, the findings from this study could be largely generalised to other states in the country. Also, the quasi-experimental design limited the ability to draw causal conclusions, and there was lack of adequate follow-up to assess the effect of decentralisation on PTLTFU during the treatment and compare the intervention and control groups.

## CONCLUSION

Our study showed that decentralised DR-TB services resulted in an improvement in the proportion of PwDRTB enrolled in treatment in Nigeria and the median time-to-treatment reduced in both intervention and control states. As a pilot, this study serves as a reference guide for the expansion of decentralised services to other parts of the country.

## Supplementary Material


