# Supplementary material for: Decentralising drug-resistant TB treatment initiation services
Source: IJTLD Open. 2025 Dec 10;2(12):757–62. doi: 10.5588/ijtldopen.25.0412 (PMC12699969; doi:10.5588/ijtldopen.25.0412)
Supplement: Supplementary file 1 [file ijtldopen25-0412_supplementarydata1.pdf]

Supplementary material

Background

The TB REACH Wave 9 project titled *"Catalyzing improvements in DR-TB care in Nigeria: A Sustainable Patient-centered approach"* was a 15-months (1st April 2022 – 30th June 2023) project grant funded by the STOP-TB Partnership and was implemented by RedAid Nigeria in two southern Nigeria States (Akwa-Ibom and Oyo). The project aimed at addressing the drug-resistant (DR-TB) diagnostic-enrolment gap by reducing pre-treatment loss to follow-up (PTLTFU) through decentralized DR-TB services.

Detailed description of interventions for decentralized DR-TB treatment initiation services

Several multi-level interventions (Figure) were strategically designed to provide patient-centred care by addressing identified gaps in the DR-TB care pathway in Nigeria from the point of laboratory confirmation of a DR-TB diagnosis, up to treatment initiation. The package of different interventions was designed to synergistically reduce PTLTFU and were implemented by the existing health workers and relevant personnel involved in the routine programmatic management of DR-TB within the intervention states.

1) *Devolving DR-TB diagnosis notification to include local government TB supervisors (TBLS) (using WhatsApp) as well as to the person with DR-TB:* Decentralizing DR-TB diagnosis notifications from the laboratories was a digital intervention designed to facilitate prompt initiation of necessary action by the responsible program staff to improve timely enrolment on treatment.
2) *Working with community-based organizations (CBOs) to improve tracing of PwDRTB through output-based financial incentives*: Through the engagement of CBO, the project aimed at increasing the proportion of diagnosed eligible patients who are tracked and linked to care.
3) *Improving counselling of people with DR-TB through introduction of structured pre-treatment counselling and team counselling with DR-TB survivors*: DR-TB survivors were trained to conduct motivating peer counselling for newly diagnosed DR-TB patients with the team of program staff. Patients were also empowered with multimedia educational materials. The objective was to reduce the proportion newly diagnosed DR-TB patients who refuse the (free) DR-TB treatment and improve retention-in-care.
4) *Decentralizing baseline investigations to more (pre-qualified) peripheral laboratories*: Prior to the intervention, there were only 1 or 2 centrally located laboratories eligible to conduct mandatory baseline investigations prior to commencing treatment. The challenge of time, travel distance, transportation cost and other opportunity cost made this a huge bottleneck delaying treatment initiation. This project intervention was aimed at reducing the proportion of persons awaiting baseline investigations by

enlisting pre-qualified (mainly) private sector owned laboratories closer to the communities to improve access and ease conducting the requisite tests.

5) *Providing transport support to PwDRTB for baseline investigations*: A financial incentive to support DR-TB patients' transportation cost to access baseline investigations and commence treatment. However, this was no longer implemented as planned because the Global Fund routine program launched a similar nationwide transport support package to bridge this gap. This validates the project's conceptualization of the drivers of PTLTFU in Nigeria.
6) *Employing mobile connectivity solutions to improve turnaround time of baseline investigations using Unstructured Supplementary Service Data (USSD)*: The USSD application was a digital intervention using a laboratory information management system to reduce the proportion of persons awaiting baseline investigations by reducing the turnaround time for retrieving baseline investigations results.
7) *Decentralizing treatment initiation to the Local government area (LGA) level*: Rather than centralized DR-TB treatment enrolment at the state capital, community-based local government TB Supervisors (TBLS) were trained and empowered to initiate newly diagnosed DR-TB patients on treatment at the communities. The last step and core of treatment enrolment decentralizing, this was possible because of the recently adopted all-oral shorter regimen.
8) *Engagement of a state (volunteer) liaison officer to coordinate state-wide activities*: The only ad-hoc staff engaged by the project (1 person per state), this role facilitates on-site coordination of DR-TB services by supporting the state and LGA teams to actively monitor, fast-track treatment enrolment activities and ensure proper documentation by accounting for every diagnosed patient.

A series of trainings for capacity building was conducted for all cadres of frontline health workers who implemented the interventions. This was followed by close, supportive supervision and mentoring throughout the implementation process.

A comprehensive documentation of the entire project is detailed in this **Technical Report**.

Interventions for decentralizing DR-TB treatment initiation services

- Devolving DR-TB diagnosis notification to include local government TB supervisors (TBLS) (using WhatsApp) as well as to the person with DR-TB
- Working with community-based organizations (CBOs) to improving tracing PwDRTB through output-based financial incentives
- Improving counselling of people with DR-TB through introduction of structured pre-treatment counselling and team counselling with DR-TB survivors
- Decentralizing baseline investigations to more (pre-qualified) peripheral laboratories
- Providing transport support to PwDRTB for baseline investigations
- Employing mobile connectivity solutions to improve turnaround time of baseline investigations using Unstructured Supplementary Service Data (USSD)
- Decentralizing treatment initiation to the LGA level
- Engagement of a state (volunteer) liaison officer to coordinate state-wide activities

Figure: List of interventions to decentralize treatment initiation for Drug-Resistant Tuberculosis
